# Simultaneous inhibition of PARP/AKT to intercept nascent *BRCA1/2*^*mut*^ breast tumors

**DOI:** 10.1038/s41523-026-00892-6

**Published:** 2026-01-16

**Authors:** Lin Wang, Brian R. Sardella, Emily K. Aronson, Zhaoji Liu, Xiaohui Li, Gerburg M. Wulf, Yujing J. Heng

**Affiliations:** 1 Department of Medicine, Beth Israel Deaconess Medical Center & Harvard Medical School, Boston, MA USA; 2 Department of Pathology, Beth Israel Deaconess Medical Center & Harvard Medical School, Boston, MA USA

**Keywords:** Cancer, Oncology

## Abstract

We utilized the *K14-Cre Brca1*^*f/f*^*Tp53*^*f/f*^ mouse model to investigate whether a pulse of PARP inhibitor (PARPi) ± an AKT inhibitor (AKTi) can prevent *Brca1*-related breast cancer. PARPi alone did not intercept or prevent tumor development. PARPi+AKTi intercepted tumors but did not prevent new tumors. These data confirm the efficacy of a PARPi and an inhibitor of PI3K signaling in treating *BRCA1/2*-related tumors, but this combination is not sufficient to prevent carcinogenesis.

Surgical and medicinal interventions are the current strategies for primary and secondary prevention of *BRCA1/2*-related cancers. Surgical interventions include bilateral prophylactic mastectomies, a highly effective but invasive procedure, and bilateral salpingo-oophorectomy, which significantly reduces ovarian cancer risk but induces premature menopause^1^. Tamoxifen lowers the risk of primary breast cancer by about 65% in *BRCA1/2*^*mut*^ carriers^2^. For secondary prevention, PARP inhibitors (PARPi), such as olaparib, have demonstrated efficacy in reducing recurrence and metastatic disease in high-risk stage II or III breast cancers^3^. However, it remains unclear whether adjuvant olaparib contributes to resistance upon later recurrence. When considering primary prevention in at-risk individuals, toxicity of the preventative treatment is a major concern. PARPi have been linked to chronic anemia and myelotoxicity, including a potential risk for myelodysplastic syndrome^4^. Therefore, the continuous administration of a PARPi to healthy women would be prohibitive, while a short, 5–7 day pulse might be tolerable and effective for prevention. The concept of cancer prevention with a pulse of a preventative agent has been previously explored^5,6^.

PARPi exert anti-tumor effects by disrupting DNA damage repair pathways in cancer cells harboring defects in homologous recombination (HRD)^7^. Beyond DNA damage repair, PARPi have been shown to enhance epithelial cell survival in nutrient-deprived and hypoxic conditions^8,9^. This effect is incompletely understood but has been variably attributed to the accumulation of the PARP substrate NAD⁺ following PARP inhibition, a moonlighting role of PARP as a transcription factor, and altered intracellular signaling resulting from the inhibition of poly(ADP-ribosyl)ation of non-nucleic acid targets^9^.

We previously published that the inhibition of glycolytic flux using a PI3K inhibitor enhanced the efficacy of PARPi in breast cancer^10–12^, supporting the hypothesis that PARPi-induced metabolic reprogramming may blunt its own therapeutic activity. This concept led to a clinical trial combining PARPi with a PI3K inhibitor (NCT01623349)^13–16^. Capivasertib, an AKT inhibitor (AKTi), has shown efficacy in tumors harboring a *PIK3CA, AKT*, or *PTEN* mutation, with a more favorable safety profile than the PI3K inhibitor, alpelisib^17^.

In this manuscript, we investigated whether a short 3- to 7-day course of combined oral PARPi and AKTi can 1) intercept nascent *BRCA1/2*^*mut*^ tumors that are too small to be detected by imaging, and 2) serve as a primary prevention strategy for at-risk *BRCA1/2*^*mut*^ carriers without radiographic evidence of malignancy. We also assessed whether this brief preventative intervention promotes the emergence of PARPi resistance.

We designed a three-arm interception study (Fig. 1A) using a genetically engineered mouse model (GEMM) *K14-Cre Brca1*^*f*^^*/f*^*Tp53*^*f/f*^^10,11,18–22^. We implanted ~1 mm^3^ of previously cryobanked *K14-Cre Brca1*^*f*^^*/f*^*Tp53*^*f/f*^ tumor fragments into mature, virgin wild type mice to mimic micro-invasive, or early-stage breast cancer, below the threshold of detection. Our engraftment rate was 80%, demonstrating the robustness of the method. Mice were treated for 5 days with a pulse of PARPi and AKTi 11 days after implantation at a time when engraftment would have occurred. The 5-day pulse treatment duration was derived using a power law relationship equation extrapolating from prior treatments of 4–6 mm tumors (Suppl 1). Endpoint for this study was tumor size of 10 mm in its largest extension. The control arm had the most number of tumors (8/10, 80%), followed by PARPi (6/10, 60%), and PARPi+AKTi (3/9, 33.3%; Fig. 1B). Animals treated with the PARPi+AKTi combination had a 58% lower risk of cancer progression compared to control (relative risk (RR) 0.42, *p* = 0.07). The PARPi+AKTi arm also had 44% lower risk of cancer progression compared to PARPi but this finding did not achieve significance (RR 0.56, *p* = 0.37). There was no significant interception with the PARPi alone compared to control (RR 0.75, *p* = 0.63). In animals that remained tumor-free, necropsy confirmed the absence of tumors at the implantation site (Fig. 1C).

**Fig. 1:**
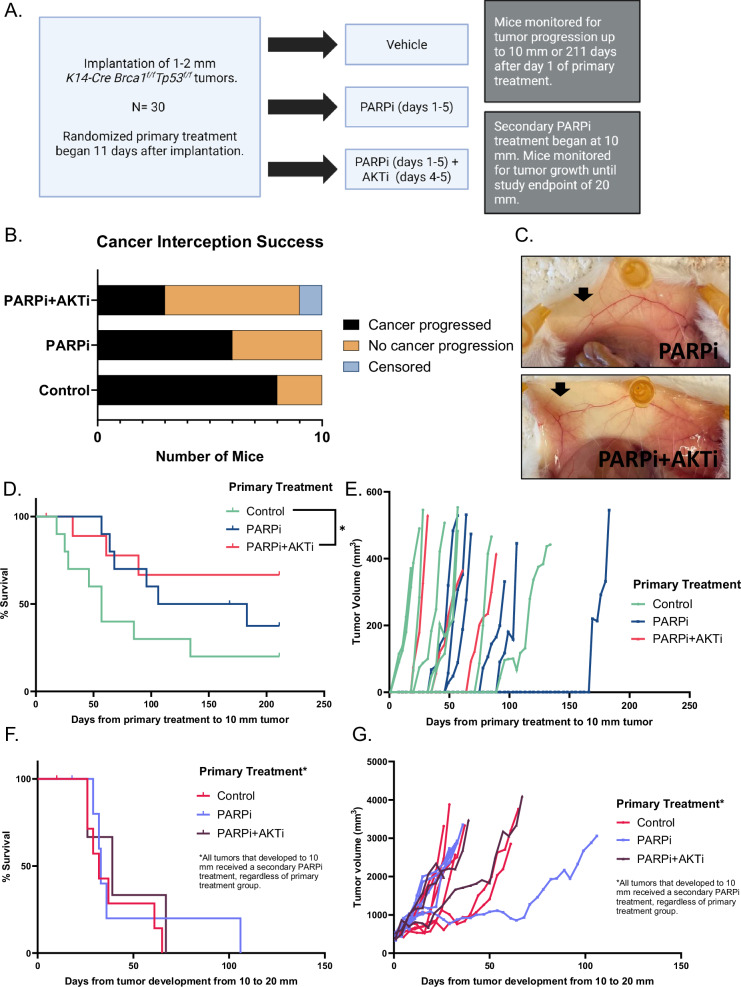
Interception study design and results. **A** Experimental design for the interception study. Eleven days after tumor implantation, mice were randomized to receive primary treatment for 5 days: 1) vehicle; 2) PARP inhibitor (PARPi; treatment days 1-5); or 3) PARPi (treatment days 1-5) and AKT inhibitor (AKTi; treatment days 4-5). The average tumor diameter at day 11 was between 2 to 3 mm. PARPi, olaparib, was administered daily 50 mg/kg i.p., while AKTi, capivasertib, was administered daily 120 mg/kg orally. **B** Cancer interception rates in the three treatment arms. **C** Example of necropsy images to confirm that the mice achieved complete interception of the tumor at the end of the experiment. Black arrows indicate the site of tumor implantation. **D** Kaplan–Meier survival curves depicting overall survival of mice across treatment groups. Tumor reaching 10 mm in the longest diameter was set as the endpoint. **E** The growth curves of the tumors from starting primary treatment to 10 mm endpoint, corresponding to (**D**). **F** Kaplan-Meier curves show the treatment outcome starting from 10 mm to 20 mm tumor diameter. **G** The growth curve of the tumors corresponding to (**F**).

The median time to development of a 10 mm tumor after the pulse primary treatment was 57 days for the controls, 145 days for PARPi and over 211 days for the PARPi+AKTi combination (Fig. 1D). However, variability was large and the delay in tumor onset was significant only for the PARPi+AKTi combination compared to controls (*p* = 0.03, log-rank test; Fig. 1D). Tumor doubling time did not differ between the three arms (*p* = 0.86; Fig. 1E), suggesting that tumor growth kinetics were not affected by the treatment. Our results show the potential of a short pulse of a PARPi+AKTi treatment to intercept emerging *Brca1*-related tumors. The early exposure of PARPi or PARPi+AKTi did not affect tumor growth kinetics.

We then tested whether prior exposure to PARPi causes PARPi resistance. Once tumors reached 10 mm in the interception study, we performed a cross-over to active treatment and administered daily PARPi treatment (Fig. 1A). Upon PARPi treatment, the growth of some tumors was initially slowed, but eventually all tumors progressed. The median time to reach this cross-over study endpoint of 20 mm did not differ between the three primary treatment arms (between 32 and 39 days, *p* = 0.71; Fig. 1F). Tumor doubling time also did not differ (*p* = 0.84; Fig. 1G). No PARPi-naïve tumors were included as controls due to the cross-over design; however, tumor responses to PARPi aligned with previous reports (Suppl 2)^10,11,20–22^. These results suggest that previous exposure to a short pulse of a PARPi did not confer resistance when the tumors were given secondary PARPi treatment.

To investigate whether PARPi or PARPi+AKTi can prevent spontaneous *BRCA*-related tumorigenesis, we used the same *K14-Cre Brca1*^*f/f*^*Tp53*^*f/f*^ tumor model in which tumors typically develop at around age 6 months^10,11,22^. Given the median delay in tumor growth of approximately 5 months observed in our interception study (Fig. 1D), we hypothesized that treatment at age 3 months might delay tumor development to 8 months and beyond. Therefore, the preventive treatment was given to mice at the age of 3 months, and they were monitored for tumor development (Fig. 2A). As the 5-day PARPi and PARPi+AKTi treatments in the interception study were incompletely effective but well tolerated (Fig. 1B and D), we decided to extend the treatment duration to 7 days in the prevention study. The median tumor-specific survival of control mice was 78 days. The median tumor-specific survival of mice treated with PARPi+AKTi (120 days) or PARPi alone (117 days) was longer than controls but highly variable, and this difference did not reach statistical significance (*p* = 0.11 and *p* = 0.33, respectively; Fig. 2B); the effect size, an improvement of about 40 days, was much smaller than in the interception study. Tumor doubling time was also not different between the three arms (*p* = 0.30), confirming that the preventative treatment did not affect growth kinetics once tumors were established.

**Fig. 2:**
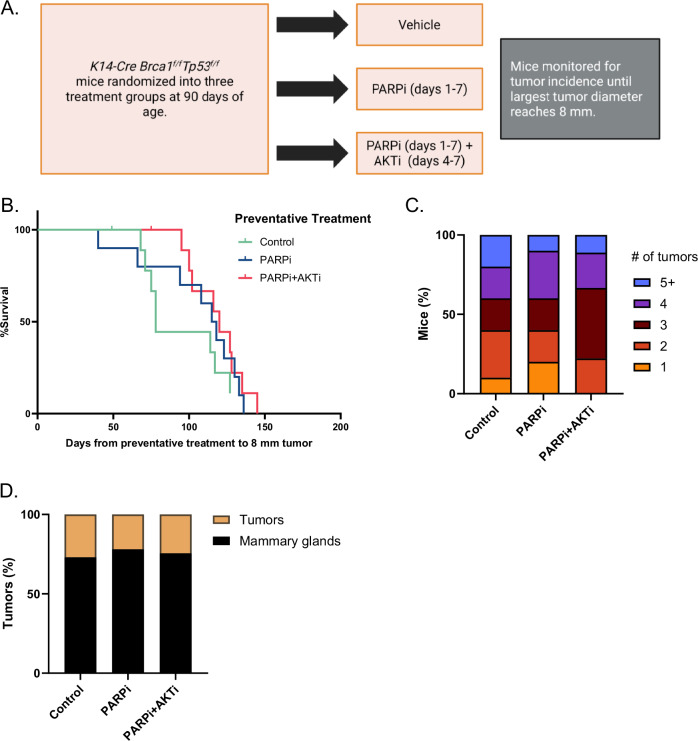
Prevention study design and results. **A** Experimental design for the tumor prevention study using the *K14-Cre Brca1*^*f/f*^*Tp53*^*f/f*^ mouse model. Mice at age of 3 months were randomized into 1) vehicle; 2) PARP inhibitor (PARPi; treatment days 1-7); or 3) PARPi (treatment days 1-7) and AKT inhibitor (AKTi; treatment days 4-7). **B** Kaplan-Meier demonstrating tumor incidences with 8 mm longest diameter of tumor was set as endpoint for single tumors or total 20 mm for multiple tumors. **C** Stacked bar graph displaying tumor burden distribution per mouse in each treatment groups. **D** Bar graph displaying tumor incidence as a percentage of mammary glands where each mice contributes 10 mammary glands.

With the exception of one mouse that was censored for unrelated death, all other mice in the prevention experiment developed at least one spontaneous tumor (Fig. 2C, D). Taken together, the 7-day preventative treatments did not reliably delay or prevent *K14-Cre Brca1*^*f/f*^*Tp53*^*f/f*^ tumors from forming.

Medicinal, primary prevention strategies for cancer have to balance the risk of taking the medication with the strength of the preventative effect. Only if that risk/benefit ratio is in favor of benefit can a clinical trial be considered. In theory, PARPi might delay or prevent tumorigenesis in cells with *BRCA1/2* mutations as they are toxic to damaged cells that might have acquired additional secondary mutations. They might also interfere with the early clonal expansion of precancerous HR-deficient cells. However, the continuous use of PARPi for years or decades is prohibitive because of its long-term toxicities, including anemia, myelodysplastic syndrome, and acute myeloid leukemia^23^.

We found that a short course (5 days) of combined PARPi and AKTi effectively intercepted the outgrowth of small, early tumors, although this was not complete as tumors continued to develop in three out of 10 mice. Surprisingly, when we applied a similar strategy to the GEMM where we expect tumors with >80% penetrance, a 7-day pulse did not prevent the emergence of new, spontaneous *BRCA1/2*^*mut*^ tumors. We saw a highly variable delay in tumor development with both PARPi alone and, more prominently, with PARPi+AKTi, but this effect did not reach significance. Since the 7-day pulse failed to prevent tumor development, we did not pursue additional experiments to shorten the treatment duration or increase its frequency. We reasoned that PARPi exposure for 5–7 days each month in mice corresponds to ~16–20% of their remaining lifespan—an unacceptably long treatment window when extrapolated to humans.

The difference between the interception and the prevention study was that a very small amount of tumor was present at the start of the pulse treatment in the interception study, while the stage of carcinogenesis was uncertain at the time of treatment in the prevention study. The failure to prevent tumor development in the GEMM was an unexpected finding because a large portion of the mammary epithelium in this model, by definition, does not express *Brca1* or *Tp53*^18^. This mimics a loss of heterozygosity situation in humans, so we expect these cells to be eliminated by PARPi. Our findings raise the possibility that additional steps towards cancerization are necessary for cells with loss of *BRCA1* to become PARPi-sensitive in vivo, and that these occur within a relatively short time frame, as most tumors after preventative PARPi or PARPi+AKTi had fully developed in the GEMM within 4 months even after the preventive pulse.

There are currently limited medical strategies for *BRCA1/2*^*mut*^ carriers who decline prophylactic surgeries, or those who are concerned about their residual cancer risk after preventive surgeries, or have completed curative treatment for prior cancers. Our findings do not support a single, short-course therapy as a strategy for prevention in *BRCA1/2*^*mut*^ carriers. However, our data suggest the possibility that a short, limited treatment of combined PARPi and AKTi could potentially intercept a low tumor burden.

Blood-based assays for circulating tumor (ct) DNA or tumor cells have shown promise in detecting minimal residual disease after cancer treatment^24^ and in early detection of cancer in asymptomatic people^25^. Currently, the clinical management for patients who test positive for ctDNA but lack otherwise evidence of disease is unclear. Our study highlights a potential application of a short-course regimen for such individuals, providing a therapeutic option in the absence of tumors detectable by imaging.

Finally, our data show that the combination of PARPi and AKTi was more effective than the PARPi alone. These data confirm our prior work showing that the combination of a PI3K inhibitor and a PARPi was more effective than PARPi alone ^10–12^. As capivasertib is more easily tolerated in combination with olaparib^26,27^, a short course of capivasertib with olaparib might be a clinically feasible interception strategy that does not cause long-term toxicity or resistance. Future studies could evaluate this regimen in a clinical setting of minimal early disease or minimal residual disease, define molecular markers of early tumor interception, optimize dosing schedules, and evaluate longer-term outcomes.

## Methods

### Animal experiments

All animal experiments were conducted in accordance with Institutional Animal Care and Use Committee (IACUC)-approved protocol at Beth Israel Deaconess Medical Center (#052-2020-23). Animals were euthanized using CO_2_ inhalation in a “Smartbox” device.

### Cancer interception study

*K14-Cre Brca1*^*f/f*^*Tp53*^*f/f*^ tumor fragments of 1 to 2 mm were implanted into the fourth mammary fat pad of 30 female FVB/NJ mice (strain#:001800; Day 0). Tumors were measured twice weekly. To determine the duration of the pulse treatment to achieve best response, we used an independent set of data and the power law relationship equation: ln(*t*)=ln(*k*)+α⋅ln(*V*) (Suppl 1). The time to best response for a 2 mm tumor was 4.43 days, hence the pulse treatment was given for 5 days. For this interception study, the endpoint was when a tumor reached 10 mm, or 211 days after the initiation of primary treatment. Our IACUC uses tumor size in its largest extension as euthanasia and endpoint criteria. The absence of a tumor was confirmed during necropsy. Cancer interception was evaluated as a means of primary treatment efficacy. Kaplan-Meier curves evaluated the median time from the start of primary treatment to the tumors reaching 10 mm. Mice with poor body condition or not reach endpoint were euthanized and censored.

### PARPi resistance study

Tumors from the interception study that reached 10 mm were given a secondary treatment of daily olaparib (50 mg/kg i.p). Mice were euthanized when their tumors reached 20 mm or 211 days after initiating primary treatment.

### Cancer prevention study

To study the effect of a preventative treatment on spontaneously developed tumors, we generated *K14-cre Brca1*^*f/f*^*Tp53*^*f/f*^ mice by crossing K14-Cre^+^ *Brca1*^*f/f*^*Tp53*^*f/f*^ males and K14-Cre^-^ *Brca1*^*f/f*^*Tp53*^*f/f*^ females and confirmed Cre+ genotype by PCR. PARPi and AKTi doses were the same as the cancer interception study. Tumor incidence or mouse health conditions were monitored. When a tumor arose, it was measured twice a week. These mice develop multiple synchronous tumors. Our IACUC stipulates that the combined largest diameter of the sum of the emergent may not exceed 20 mm. Therefore, the endpoint of this study was defined as when the largest tumor reached 8 mm in longest diameter to be safe to not exceed the IACUC-stipulated maximum tumor burden or, if multiple tumors arise, when the combined tumor burden reaches 20 mm.

### Data analysis

Pair-wise comparisons of cancer interception was performed using Fisher’s exact test in GraphPad prism v10.5.0. Tumor-specific survival was evaluated using Kaplan-Meier curves and log rank test. Tumor doubling time was calculated using the formula: (*t* x log_10_(2))/(log_10_(V_*t*_/V_*0*_)), where V_*t*_ is the volume at time *t* and V_*0*_ is the volume at *t* = 0. One-way ANOVA was used to compare tumor doubling times between the groups. Statistical significance was achieved when *p* < 0.05. Flowcharts were created using BioRender. Heng, J. (2026) https://BioRender.com/qd2eib5. Graphs were created using GraphPad prism.

## Supplementary information

Supplementary Information

## Data Availability

All data supporting the findings of this study are available within the paper and its Supplementary Information.
