# Supplementary material for: Simultaneous inhibition of PARP/AKT to intercept nascent BRCA1/2mut breast tumors
Source: NPJ Breast Cancer. 2026 Jan 16;12:27. doi: 10.1038/s41523-026-00892-6 (PMC12920871; doi:10.1038/s41523-026-00892-6)
Supplement: Supplementary file 1 — Supplementary Information [file 41523_2026_892_MOESM1_ESM.pdf]

Supplemental Figure 1.

Dataset to calculate duration of best response for a 2 mm tumor using the power law relationship.

**Diameter, *D* (mm)**	**Volume, *V* (mm^3)***	***t* (days)**	**ln(*V*)**	**ln(*t*)**
5.92	108.8	19	4.69	2.94
5.43	84.1	26	4.43	3.26
5.20	73.9	15	4.30	2.71
6.46	141.1	26	4.95	3.26
5.57	90.2	19	4.50	2.94
5.66	95.2	22	4.56	3.09
5.81	102.8	22	4.63	3.09
5.90	107.6	19	4.68	2.94

*Tumor volume was calculated using the ellipsoid formula, $V = (4/3) \times \pi \times (D/2)^3$

Power law equation: $\ln(t)=\ln(k)+\alpha\cdot\ln(V)$, where t = time to best response and V= tumor volume.

Estimated slope, $\alpha = 0.487$ (SE = 0.331)

Estimated intercept, $\ln(k) = 0.793$, $k = 2.21$

The resultant equation is: $\ln(t)=0.793 + 0.487\cdot\ln(V)$

For a 2 mm tumor, volume calculated using the ellipsoid formula will be 4.19 mm^3

$$\ln(t) = 0.793 + 0.487\cdot\ln(4.19)$$
$$\ln(t) = 1.49$$
$$t = 4.44 \text{ days}$$

Therefore, a period of 5 days was chosen for the interception study.

Supplemental Figure 2.

All tumor-bearing mice from the interception study crossed over to the resistant study, as such a PARPi-naïve control arm was not included.

To demonstrate that these *K14-Cre Brca1*$^{f/f}$*Tp53*$^{f/f}$ tumors remained responsive to secondary PARPi treatment and that prior PARPi exposure did not confer resistance, we present data from an independent study using the same tumor model. In this experiment, olaparib-treated tumors had a median tumor-specific survival of 98 days compared to 17 days in untreated controls (p<0.001; see **A** and **B** below).

Although these data are from a separate experiment, the magnitude and kinetics of PARRi response are consistent with prior reports [10,11,20-22]: untreated *K14-Cre Brca1*$^{f/f}$*Tp53*$^{f/f}$ tumors tend to progress from 5 to 20 mm in their largest extension around 14 days while olaparib-treated tumors require approximately 90–100 days to reach the same size.

Given the consistently large therapeutic effect of olaparib in this tumor model and the rapid growth of untreated tumors—from 10 to 20 mm within an average of 10 days—the Kaplan-Meier curves in Fig. 1F reflect the expected activity of olaparib. Collectively, our data support the conclusion that prior PARPi exposure did not confer resistance to secondary PARPi treatment.

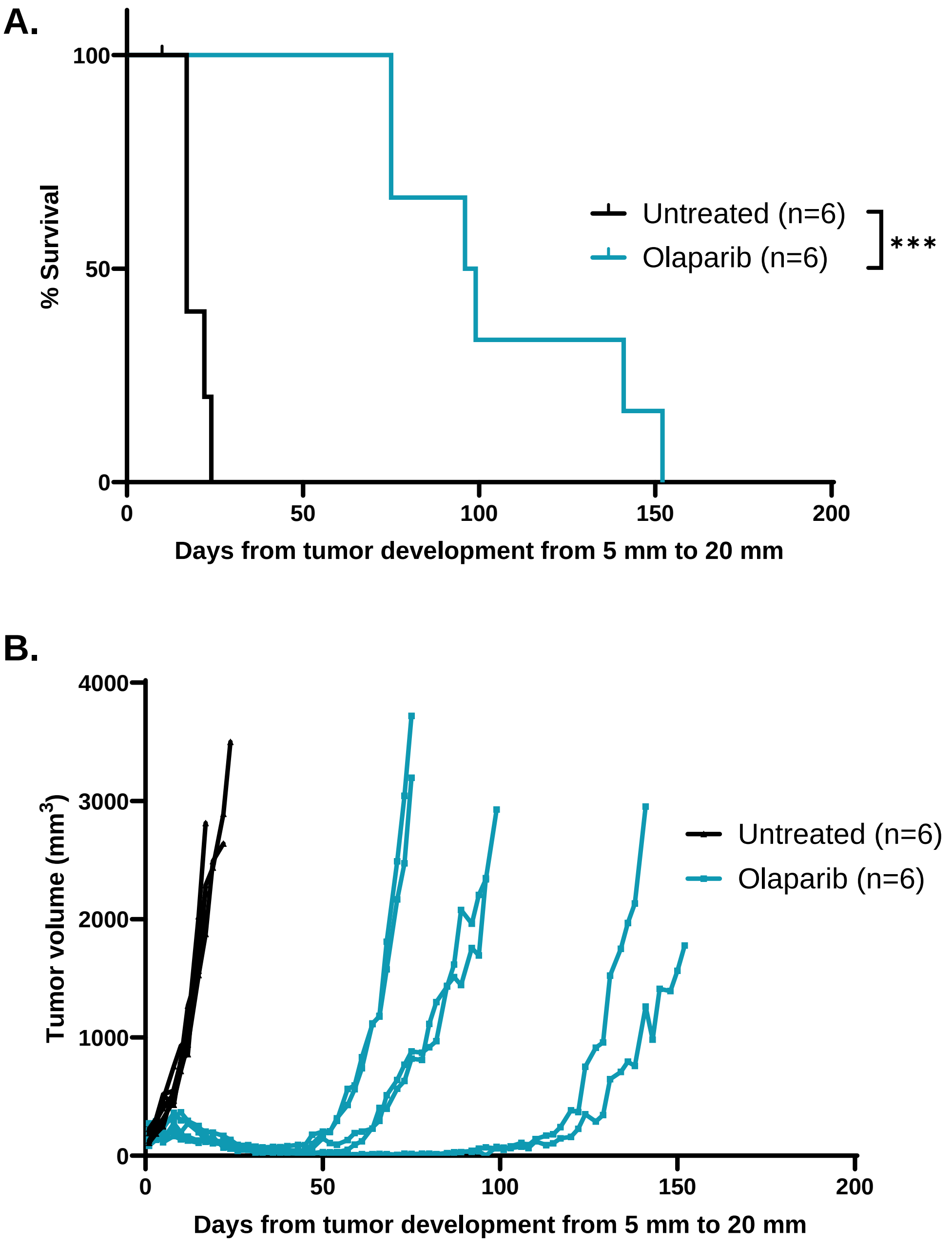

A. *K14-Cre Brca1$^{f/f}$Tp53$^{f/f}$* tumors treated with olaparib had a median tumor-specific survival of 98 days while untreated controls had a median tumor-specific survival of 17 days (p<0.001). **B.** Growth curves of the tumors in **A**.
